# The pharmacology of gepants in migraine: A scoping review on mechanisms, clinical applications, and combination strategies

**DOI:** 10.1111/head.70114

**Published:** 2026-06-09

**Authors:** Luigi Francesco Iannone, Marcello Silvestro, Antonio Munafò, Marina Romozzi, Pierangelo Geppetti, Antonio Russo

**Affiliations:** ^1^ Department of Biomedical, Metabolic and Neural Sciences University of Modena and Reggio Emilia Modena Italy; ^2^ Inter‐Departmental Program of Headache Medicine and Primary Pain Syndromes, Department of Medical, Surgical, Neurological, Metabolic and Aging Sciences University of Campania “Luigi Vanvitelli” Naples Italy; ^3^ Department of Neurosciences, Psychology, Drug Research and Child Health University of Florence Florence Italy; ^4^ Dipartimento Universitario di Neuroscienze Università Cattolica del Sacro Cuore Rome Italy; ^5^ Neurologia, Dipartimento di Neuroscienze, Organi di Senso e Torace Fondazione Policlinico Universitario Agostino Gemelli IRCCS Rome Italy; ^6^ Section of Clinical Pharmacology and Oncology, Department of Health Sciences University of Florence Florence Italy

**Keywords:** calcitonin gene‐related peptide, drug‐induced liver injury, gepants, migraine, pharmacokinetic, pharmacology

## Abstract

**Background:**

Calcitonin gene‐related peptide (CGRP) is a key mediator in migraine pathophysiology, and the development of gepants, novel CGRP receptor antagonists, has expanded therapeutic options for both acute and preventive treatment. This scoping review aims to synthesize current evidence on the pharmacology, clinical applications, safety, and combination strategies of gepants.

**Methods:**

A scoping review was conducted following PRISMA‐ScR recommendations. A comprehensive search of PubMed/MEDLINE and Scopus was performed from database inception to July 2025, with an additional search in January 2026 for real‐world studies. Data were extracted and summarized narratively without quantitative synthesis.

**Results:**

Second‐ and third‐generation gepants, including ubrogepant, rimegepant, atogepant, and zavegepant, overcome the hepatotoxicity and bioavailability limitations of earlier compounds. Pharmacokinetic properties determine their suitability for both acute and preventive use, and their lack of vasoconstrictive activity makes them suitable for patients with cardiovascular comorbidities. Most are metabolized by cytochrome 3A4 and are substrates for efflux transporters, necessitating awareness of drug–drug interactions, although no clinically significant interactions with common migraine preventive medications have been reported. Preclinical and preliminary clinical evidence indicates a low likelihood of gepants inducing medication‐overuse headache. Combination therapy with other migraine treatments is mechanistically plausible and supported by early pharmacokinetic and safety data, though robust clinical trial evidence remains limited.

**Conclusion:**

Gepants represent an effective and well‐tolerated class of CGRP‐targeted therapies with flexible use in acute and preventive migraine management. Their pharmacological properties support individualized treatment strategies and potential combination approaches; however, long‐term safety, optimal positioning, and the efficacy of combination regimens require further investigation.

## Plain Language Summary

Migraine is a highly disabling neurological disorder, and gepants represent a new class of drugs blocking a key pathway involved in migraine attacks. In this scoping review, we examined research on how gepants work, their drug properties, safety, and how they might be used alongside other migraine treatments. The results support gepants as effective, well‐tolerated options for both treating and preventing migraine, with growing evidence that they work well alone or combined with other treatments.

**Abbreviations**
AHS: American Headache Society
ALT: alanine aminotransferase
AM2: amylin 2 receptor
AMY1: amylin 1 receptor
AUC: area under the curve
BBB: blood–brain barrier
BCRP: breast cancer resistance protein
BoNT‐A: onabotulinumtoxinA
BSEP: bile salt export pump
CGRP: calcitonin gene‐related peptide
CLcr: creatinine clearance
CLR: calcitonin receptor‐like receptor
CM: chronic migraine
Cmax: maximum plasma concentration
CNS: central nervous system
CSF: cerebrospinal fluid
CYP: cytochrome P450
DILI: drug‐induced liver injury
EM: episodic migraine
EMA: European Medicines Agency
FDA: Food and Drug Administration
IC50: half maximal inhibitory concentration
IHS: International Headache Society
Ki: inhibition constant
mAbs: monoclonal antibodies
MMDs: monthly migraine days
MOH: medication‐overuse headache
NICE: National Institute for Health and Care Excellence
NTCP: sodium taurocholate cotransporting polypeptide
OATP1B3: organic anion transporting polypeptide 1B3
ODT: orally disintegrating tablet
P‐gp: P‐glycoprotein
pKB: binding affinity constant
PNS: peripheral nervous system
RAMP1: receptor activity‐modifying protein 1
RCTs: randomized controlled trials
RO: receptor occupancy
Tmax: time to maximum plasma concentration
Vd: volume of distribution

## INTRODUCTION

Migraine is a highly prevalent and disabling neurovascular disorder, affecting approximately 15% of the global population and ranking among the leading causes of years lived with disability worldwide, particularly in adults under 50 years of age.^1^ It is characterized by recurrent attacks of moderate‐to‐severe headache, often accompanied by neurovegetative symptoms such as nausea, vomiting, and sensory hypersensitivity with photophobia and phonophobia.^2^ The pathophysiology of migraine attacks is multifactorial, involving altered sensory processing leading to the activation of the trigeminovascular system with the release of several neuropeptides.^2^, ^3^

Among these, calcitonin gene‐related peptide (CGRP) has emerged as a central mediator in migraine pathophysiology.^4^ CGRP is a 37 amino acid neuropeptide widely distributed in the central and peripheral nervous systems (CNS and PNS, respectively), with particularly high concentrations in dorsal root and trigeminal ganglion neurons.^5^, ^6^ During a migraine attack, CGRP, released from perivascular nerve terminals, binds mainly to the canonical CGRP receptor, a heterodimer composed of the calcitonin receptor‐like receptor (CLR) and the receptor activity‐modifying protein 1, expressed on vascular smooth muscle cells, neurons, and satellite glial and Schwann cells.^7^, ^8^, ^9^ Other receptors potentially targeted by CGRP, including amylin and adrenomedullin, have been proposed to impact migraine pathophysiology.^2^, ^4^, ^10^ Of the two forms of CGRP found in mammals, the α‐CGRP is expressed in a subset of primary sensory neurons with C‐ and Aδ‐fibers, whereas the β‐CGRP isoform is present in intrinsic neurons of the gastrointestinal tract.^11^ CGRP engagement with its receptor results in vasodilatation, particularly in small diameter arterioles, and produces a marked sensitization of nociceptors to mechanical stimuli.^8^, ^12^ Pro‐algesic stimulation by CGRP of trigeminal and cervical sensory fibers conveys pain signals via the trigeminocervical complex to CNS projections.

The identification of CGRP as a critical effector in migraine pathogenesis led to the development of targeted therapeutics. The first agents to reach the clinic were monoclonal antibodies (mAbs) directed against either CGRP itself (fremanezumab, galcanezumab, eptinezumab) or the CGRP receptor (erenumab). These drugs demonstrated robust efficacy and safety in migraine prevention, in both randomized clinical trials (RCTs) and real‐world studies.^4^, ^6^, ^13^ In parallel, small‐molecule CGRP receptor antagonists, labeled as gepants, were developed to provide nonparenteral (orally and intranasally) administered drugs with shorter half‐lives, both for acute (rimegepant, zavegepant, and ubrogepant) and preventive (atogepant and rimegepant) indications.^14^, ^15^, ^16^, ^17^ The first generation of gepants (e.g., telcagepant, olcegepant) showed promising efficacy but were discontinued due to hepatotoxicity or poor oral bioavailability.^5^ Second‐generation gepants have overcome these limitations through structural optimization, improving metabolic stability and reducing off‐target toxicity. Currently, four gepants have received regulatory approval in major markets: ubrogepant (acute), rimegepant (acute and preventive), atogepant (preventive), and zavegepant (acute).^4^ Each drug exhibits unique pharmacokinetic and pharmacodynamic characteristics that might inform its optimal clinical use, drug–drug interaction profile, and potential for combination therapy with other migraine treatments.

The present scoping review provides an updated synthesis of gepant pharmacology, integrating preclinical and clinical data to compare the approved drugs. In the following sections, we characterized the overall pharmacology of gepants as therapeutic drugs, then the pharmacological features of single gepants and the differences with first generation gepants, their permeability to the blood–brain barrier (BBB), and their potential role in inducing medication‐overuse headache (MOH). Finally, we reviewed the potential for combining gepants and other acute and preventive drugs for migraine and current evidence of gepants positioning according to guidelines.

## METHODS

This study was conducted as a scoping review reported following the Preferred Reporting Items for Systematic Reviews and Meta‐Analyses extension for Scoping Reviews. A scoping review methodology was chosen to map the pharmacology of gepants, including their mechanisms of action, pharmacokinetics, clinical applications, and potential for combination strategies in migraine treatment, without restricting inclusion based on study quality or performing quantitative synthesis. No *a priori* protocol was registered.

### Information sources and search strategy

A comprehensive literature search was performed in PubMed/MEDLINE, and Scopus from beginning to July 2025. The search strategy combined terms related to gepants (“ubrogepant,” “rimegepant,” “atogepant,” “zavegepant,” “telcagepant,” and “MK‐3207”) with “CGRP receptor antagonist,” “migraine,” “pharmacology,” “pharmacodynamic,” and “pharmacokinetics.” Following database‐specific Boolean strategies and the Scopus search was conducted using the “TITLE‐ABS‐KEY” fields. Reference lists of included studies and relevant reviews were screened to capture additional studies.

Two reviewers (MR and LFI) independently screened articles titles and abstracts for relevance. Full texts of potentially eligible articles were retrieved and assessed against the inclusion criteria above.

Given the heterogeneity of study designs and outcomes, no quantitative synthesis was performed and results were summarized narratively and organized by drug molecule and into thematic paragraphs.

An additional search was conducted in January 2026 focused on real‐world studies with gepants.

The flowchart of studies is reported in Figure S1.

### Eligibility criteria

Studies were selected according to the population–concept–context framework recommended for scoping reviews.

#### Population

Human participants with migraine and related healthy controls and relevant animal or *in vitro* models used to investigate gepants pharmacology.

#### Concept

Pharmacology of gepants, including receptor binding, pharmacokinetics, metabolism, safety and hepatotoxicity mechanisms, BBB permeability, MOH risk, and combination strategies.

#### Context

Preclinical studies, clinical trials (phase 1–4), pharmacokinetic/pharmacodynamic studies, and regulatory or translational research.

Eligible publications included randomized controlled trials, open‐label studies, observational cohorts, mechanistic *in vitro* or *in vivo* studies, narrative and systematic reviews, and conference abstracts. No restrictions were applied regarding comparator, duration of follow‐up, or outcome measures. Only articles published in English were included. All Food and Drug Administration (FDA) and European Medicines Agency pharmacological reports on gepants were selected and included separately.

## GEPANTS PHARMACOLOGICAL OVERVIEW

The therapeutic application of gepants is determined by the interplay between their pharmacodynamic potency, receptor selectivity, and pharmacokinetic properties. All currently approved gepants act as potent, reversible, competitive antagonists at the canonical CGRP receptor, with inhibitory constants (*K*_i_) in the low nanomolar range.^4^ This high affinity ensures effective blockade of CGRP‐mediated signaling in migraine‐sensitive areas at clinically achievable plasma concentrations. Some agents, notably rimegepant and atogepant, exhibit measurable antagonism at the amylin 1 (AMY1) receptor, which is a heterodimer of CLR and RAMP3.^18^, ^19^ Although the clinical significance of AMY1 antagonism remains under investigation, it may contribute to broader modulation of CGRP‐evoked pain processing.

**FIGURE 1:**
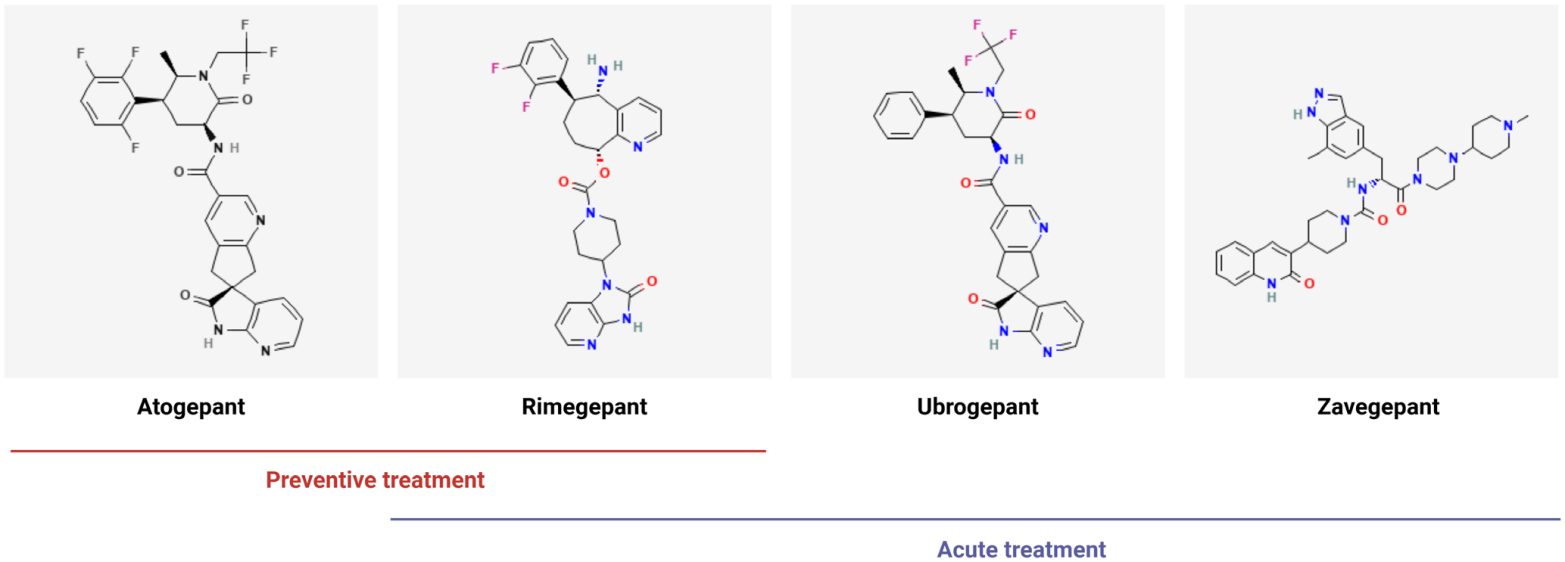
Molecular structure of gepants and their indication as acute or preventive treatment. [Color figure can be viewed at wileyonlinelibrary.com]

Gepants for acute use (ubrogepant, rimegepant, and zavegepant) are formulated and dosed to achieve rapid plasma peak concentrations (*C*_max_) and high receptor occupancy within 1–2 h of administration,^14^, ^15^, ^16^, ^20^ aligning with the therapeutic window for aborting migraine attacks. The relatively short elimination half‐life of ubrogepant (5–7 h) and zavegepant (6–7 h) limits their suitability for preventive therapy.^14^, ^15^, ^20^ In contrast, gepants used for prevention (atogepant and rimegepant) maintain therapeutic plasma levels over extended periods to provide continuous receptor blockade without excessive dosing frequency. Both have elimination half‐lives of ~11 h, permitting once daily dosing for atogepant^21^ and every‐other‐day dosing for rimegepant in preventive treatments.^16^ Steady‐state concentrations are reached within a few days, and dose‐exposure relationships are approximately linear across therapeutic ranges.^14^, ^15^, ^16^, ^17^ Molecular structures of gepants are reported in Figure 1.

Most gepants are metabolized predominantly by cytochrome P450 3A4 (CYP3A4) and are substrates for efflux transporters such as P‐glycoprotein (P‐gp) and breast cancer resistance protein (BCRP).^14^, ^15^, ^16^, ^17^ These properties necessitate consideration of potential pharmacokinetic drug–drug interactions, particularly with strong CYP3A4 inhibitors or inducers. Clinical pharmacology studies have generally demonstrated no significant interactions including oral contraceptives, triptans, topiramate, or anti‐CGRP monoclonal antibodies (mAbs), supporting their potential integration into multi‐drug regimens when clinically indicated.^22^, ^23^, ^24^, ^25^, ^26^, ^27^, ^28^, ^29^, ^30^, ^31^, ^32^, ^33^, ^34^ The differentiation between acute and preventive gepant use is not absolute: the dual approval of rimegepant illustrates that with appropriate half‐life and tolerability, a single gepant can be effectively deployed in both contexts. This versatility is likely to influence future development strategies. Indeed, an ongoing study is investigating the role of atogepant as acute treatment (ECLIPSE study, NCT06241313).^35^ An overview of the pharmacological features of gepants is reported in Table 1.

**TABLE 1:** Pharmacological features of gepants.

	Atogepant	Rimegepant	Ubrogepant^a^	Zavegepant^a^
Indication in migraine	Preventive in episodic and chronic migraine	Preventive in episodic migraine and acute	Acute	Acute
Formulation/route	Oral tablet	Oral disintegrating tablet	Oral tablet	Intranasal spray
Dosage	10, 30, or 60 mg daily^b^	Acute: 75 mg as needed (max 75 mg/day) Preventive: 75 mg every other day	50–100 mg (max 200 mg/day)^c^	10 mg (max 10 mg/day)
Molecular weight (Da)	603.5	534.5	549.5	675.2
*K*_i_ (nM)	~0.026	~0.027	~0.07	~0.023
cLog P	4.12	3.05	3.12	3.11
Peak serum concentration (*C*_max_)	740 ng/mL (60 mg)	907.5 ng/mL (75 mg)	117 ng/mL (100 mg)	13.4 ng/mL
Time‐to‐peak plasma concentration (*T*_max_), h	1.5	1.5	1.5	0.5
Bioavailability	~62%	~64%		~5%
Volume of distribution	292 L	120 L	350 L	1774 L
Elimination half‐life (h)	~11	~11	5–7	6–7
CYP for metabolism	CYP3A4 (major)	CYP3A4 (major), CYP2C9 (minor)	CYP3A4 (major)	CYP3A4 (major), CYP2D6 (minor)
Plasma protein binding %	~95%	~96%	~87%	90%
Clearance	19 L/h	8.9 L/h	87 L/h	266 L/h

*Note*: cLog P (partition coefficient) that measures the drug's lipophilicity, useful for predicting membrane permeability; log S that measures the drug's water solubility, important for oral bioavailability; receptor affinity (CGRP) refers to the inhibition constant (*K*_i_). *K*_i_ is a measure of the binding affinity of an inhibitor for its target, representing the concentration of the inhibitor required to produce half of the maximum inhibition. Lower *K*_i_ values indicate higher affinity. Values are expressed in pM (picomolar) if not otherwise specified.

Abbreviations: CYP, cytochrome; Da, Dalton.

^a^ Ubrogepant and zavegepant are approved in United States but not in Europe.

^b^ The 30 mg dose is not available in Europe.

^c^ The tablets are available in 50 and 100 mg.

It should be noted that the majority of available pharmacokinetic and pharmacodynamic data for gepants (reported above and in the specific paragraphs below) derive mainly from single‐dose or short‐term studies conducted predominantly in healthy volunteers under controlled conditions. Although these studies are essential for characterizing pharmacokinetic properties, they may not fully capture exposure‐response relationships in patients with migraine, particularly during repeated or long‐term dosing, during acute migraine attacks, or in the presence of important comorbidities, or polypharmacy.

In addition, pharmacodynamic inferences are often extrapolated from receptor affinity, plasma concentration‐time profiles, or surrogate biomarkers rather than from direct clinical end points, limiting the ability to precisely define concentration‐effect thresholds relevant to efficacy or tolerability. Importantly, the absence of head‐to‐head randomized controlled trials directly comparing different gepants precludes definitive conclusions regarding relative efficacy, onset of action, durability of response, or safety profiles across agents.

### Evolution of gepants across generations

The development of CGRP receptor antagonists has advanced through different development generations, moving from proof‐of‐concept intravenous agents to orally available molecules.^36^, ^37^ The first compound, olcegepant (BIBN4096BS), confirmed the role of CGRP in migraine showing efficacy as acute treatment.^38^, ^39^ However, its unfavorable physicochemical profile, high molecular weight, polarity, and multiple hydrogen‐bond donors, resulted in poor oral absorption and reliance on intravenous delivery, preventing further development.

Therefore, the next phase of development tested only oral administered molecules. Telcagepant (MK‐0974) demonstrated efficacy as acute migraine treatment^40^ and was also investigated for prevention^41^ and peri‐menstrual dosing.^42^ Although intermittent acute use appeared safe, chronic or intensive dosing caused dose‐dependent drug‐induced liver injury (DILI) with alanine aminotransferase (ALT) elevations several times (up to >10‐fold) above the upper limit of normal, with most elevations occurred between 4 and 6 weeks of treatment and none associated with concomitant elevation of total bilirubin.^40^, ^41^, ^42^

Another oral gepant known as MK‐3207, although structurally distinct, showed the same issue.^36^, ^43^ Hepatotoxicity was attributed to metabolic activation of its aniline substructure and formation of reactive phenyl–glyoxal intermediates. Different from telcagepant, abnormalities generally occurred weeks after patients had stopped taking treatment and in some cases required immunosuppressive treatment.^43^

**FIGURE 2:**
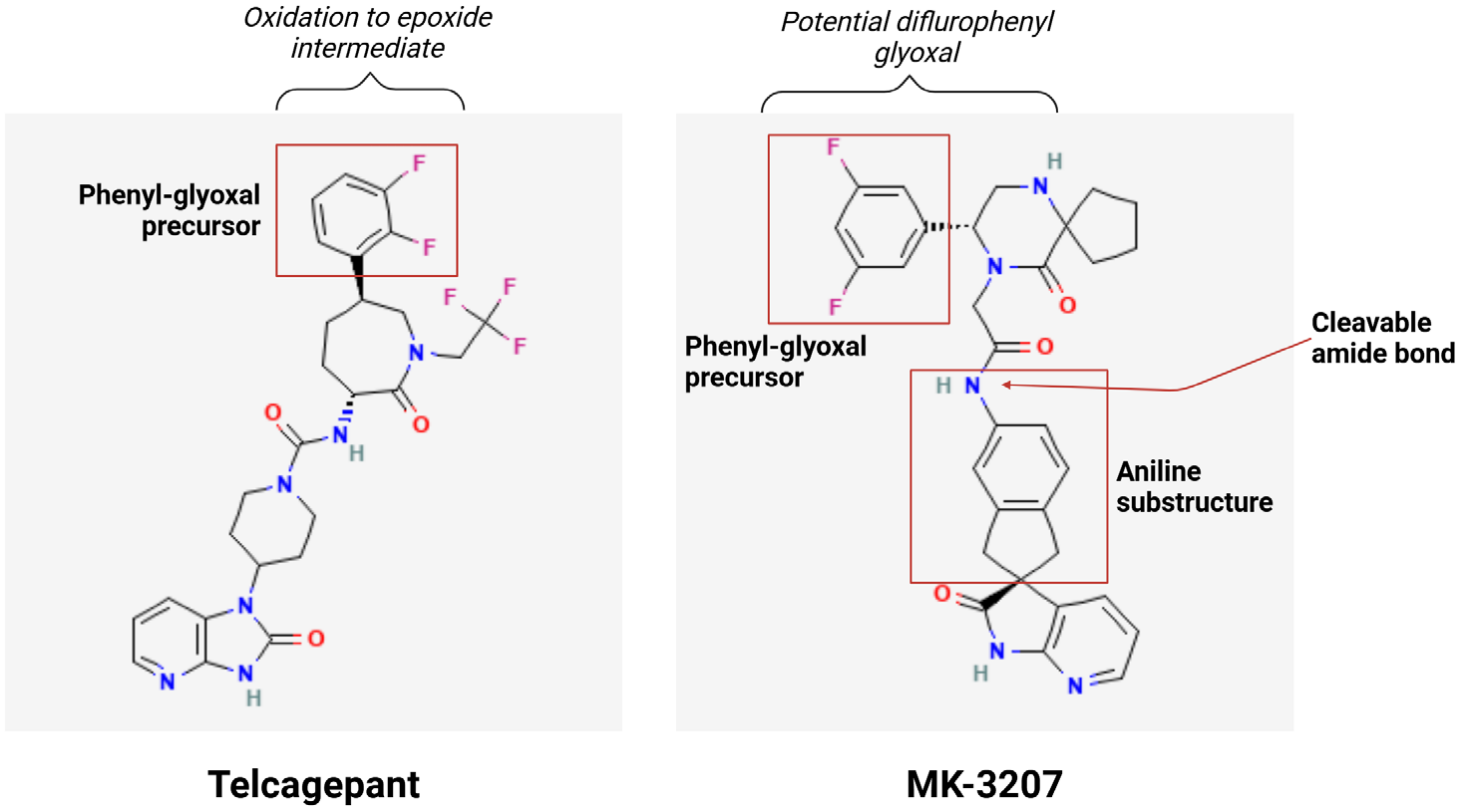
Molecular structure of first generation gepants (telgacepant and MK‐3207, not commercially available) with highlighted the potential reactive metabolite pathways (in italics) and key structural differences responsible for hepatotoxicity (red squares and arrows). Data reported in Hargreaves and Olesen^37^ and Smith et al.^44^ [Color figure can be viewed at wileyonlinelibrary.com]

Therefore, reactive metabolite formation was mainly due to oxidative metabolism of electron‐rich aromatic systems (difluorophenyl in telcagepant and aniline in MK‐3207). These groups were highly susceptible to CYP450‐mediated hydroxylation or oxidation, leading to unstable electrophilic intermediates (quinone–imines and phenyl–glyoxals) capable of covalently binding proteins in hepatocytes, triggering DILI (Figure 2).

At the same time, mechanistic studies and genetic evidence from α‐CGRP knockout mice, which do not develop liver dysfunction, and the absence of hepatotoxic signals with anti‐CGRP mAbs^45^ suggested that the responsibility was compound‐specific rather than target‐ or class‐driven.^36^, ^37^

Considering the preliminary effectiveness of gepants, the development continued, with medicinal chemistry efforts focused on eliminating chemical motifs prone to reactive metabolite formation. The result was the second generation of gepants, led by ubrogepant and atogepant.^15^, ^17^ Both molecules were structurally optimized to improve lipophilicity, metabolic stability, and oral bioavailability while avoiding hepatotoxic liabilities.^18^, ^20^ Specifically, they lack the difluorophenyl and aniline moieties associated with oxidative bioactivation in earlier compounds. Instead, their scaffolds incorporate fluorinated heteroaryl groups and constrained bicyclic cores that reduce susceptibility to cytochrome P450 mediated oxidation. These changes markedly lower the risk of forming electrophilic intermediates capable of covalent protein binding. In parallel, these compounds have optimized hydrogen‐bond donor counts and reduced molecular weight, balancing solubility and permeability to ensure reliable oral delivery. Details about chemical differences among gepants are reported in Hargreaves and Olesen,^37^ Yasuda et al.,^46^ Woodhead et al.,^47^ and Smith et al.^44^

To explore the differences among first‐ and second‐generation gepants, a quantitative systems toxicology platform, DILIsym v6A, which integrates physiologically based pharmacokinetic models with mechanistic *in vitro* data on bile acid transporter inhibition, mitochondrial dysfunction, and oxidative stress, was used.^47^ For each gepant, *in vitro* assays were performed to determine IC_50_ values for bile salt export pump (BSEP) inhibition, effects on mitochondrial electron transport chain (ETC), and propensity to induce reactive oxygen species. These results were supplied into virtual patient populations to simulate liver exposure and predict the incidence and severity of ALT and bilirubin elevations. Telcagepant simulations correctly reproduced the observed hepatotoxicity, predicting clinically meaningful enzyme elevations at therapeutic exposures. In contrast, simulations for rimegepant, ubrogepant, atogepant, and zavegepant predicted minimal risk, even when worst‐case exposure scenarios were modeled.^47^

Mechanistic dissection within DILIsym highlighted why telcagepant behaved differently.^47^ Its hepatotoxicity occurred from a combination of ETC inhibition and mixed‐mode BSEP inhibition, a dual mechanism known to strongly predict severe DILI. By contrast, newer gepants displayed only a single dominant liability, such as reactive oxygen species formation or mild ETC inhibition, which could be countered by adaptive responses such as mitochondrial biogenesis or antioxidant pathways. Moreover, telcagepant mixed inhibition of BSEP meant that bile salt export was impaired regardless of intracellular bile acid levels, leading to accumulation and hepatocyte injury. In contrast, rimegepant acts as a competitive BSEP inhibitor, which allows export activity to recover as substrate concentration increases. Importantly, modeled liver concentrations of rimegepant ensure preservation of bile acid homeostasis.^47^

In an earlier study,^44^ DILIsym was applied to assess telcagepant and ubrogepant alongside MK‐3207. That analysis predicted hepatotoxicity for telcagepant and MK‐3207, whereas ubrogepant was projected to be safe.^44^ Despite minor differences in outcomes between the two models, the consistent qualitative conclusion, that telcagepant posed a risk of severe liver injury whereas ubrogepant did not, underscores the robustness of the DILIsymu2019s quantitative systems toxicology framework.

Importantly, these predictions were made prospectively, before phase 3 clinical data were available, and were later validated in large, randomized trials where no clinically significant hepatotoxicity emerged.^4^, ^45^ At approved doses, rimegepant, ubrogepant, and atogepant showed no hepatotoxicity warnings on their labels, and the incidence of ALT elevations in trials was low, transient, and unaccompanied by bilirubin abnormalities, with no clinically meaningful hepatotoxicity even with repeated dosing and long‐term treatment.

### Atogepant

Atogepant is an oral, second‐generation CGRP receptor antagonist developed and approved for the preventive treatment of episodic and chronic migraine.^17^ It was designed to maximize oral bioavailability, extend receptor occupancy, and minimize off‐target interactions. It is approved by FDA in three dosages 10, 30, and 60 mg once daily.^48^ In Europe, only 10 and 60 mg dosages are available.^49^ In radioligand binding studies, atogepant exhibits subnanomolar affinity for the human CGRP receptor (*K*_i_ ≈ 0.026 nM) and weaker antagonism at the AMY1 (*K*_i_ ≈ 2.40 nM) receptor without affecting other neurotransmitter receptors such as calcitonin, adrenomedullin, and other neurotransmitter receptor targets.^18^, ^50^

Pharmacokinetically, atogepant has an absolute oral bioavailability exceeding 60%, with a median time to maximum plasma concentration (*T*_max_) of 1–2 h under fasting conditions. The terminal elimination half‐life is approximately 11 h, allowing for once‐daily dosing.^18^, ^21^, ^50^ When administered with a high‐fat meal, no clinically significant food effect was observed (area under the curve [AUC] and *C*_max_ decreased by approximately 18% and 22%, respectively); however, the median time to reach peak concentration remained unchanged.^51^ At therapeutic doses, steady‐state plasma concentrations are achieved within 2–3 days, and pharmacokinetics are linear over the clinical dose range.^18^, ^21^, ^50^ Atogepant undergoes extensive hepatic metabolism, primarily via cytochrome CYP3A4, with minor involvement of CYP2D6 and glucuronidation. It is also a substrate for P‐gp and BCRP, although transporter‐mediated effects on exposure are secondary to CYP3A4 metabolism. Excretion occurs mainly via feces (~42% unchanged drug) and minimally via urine (~5%).^18^, ^21^, ^50^ To note, its pharmacokinetic profile remains linear at doses up to 170 mg, nearly 3‐fold higher than the highest recommended dose for migraine prevention.^18^, ^21^, ^50^ See Table 1 for details. Pharmacokinetics are generally consistent across different patient populations, including those with varying age, sex, body weight, and mild or moderate hepatic impairment. However, dose adjustments (10 mg) are recommended for patients with severe renal impairment (creatinine clearance <30 mL/min) or end‐stage renal disease.^18^, ^21^, ^48^, ^50^

Drug–drug interaction studies have demonstrated that coadministration with the strong CYP3A4 inhibitor itraconazole increases systemic exposure by ~2.2‐fold, necessitating dose reduction, whereas the strong inducer rifampin reduces exposure by approximately 60%, potentially reducing efficacy.^18^, ^21^, ^48^, ^50^ Therefore, when coadministered with strong CYP3A4 inhibitors, the daily dose should be reduced to 10 mg. In the presence of strong or moderate CYP3A4 inducers, higher doses of 30 mg or 60 mg once daily are advised to maintain therapeutic levels.^48^, ^49^, ^50^ Atogepant has a favorable tolerability profile. The most frequent adverse events in clinical trials were constipation (6%–8%), nausea (4%–6%), and fatigue (~4%), typically mild to moderate in severity. Importantly, no hepatotoxicity signal was detected in clinical studies up to 52 weeks, and no laboratory monitoring is required in routine practice.^17^, ^21^

### Rimegepant

Rimegepant is the only gepant currently approved for both acute and preventive treatment of a migraine attack. The drug is formulated as a 75 mg orally disintegrating tablet every‐other‐day for prevention in episodic migraine and as needed for acute treatment.^16^ It binds the human CGRP receptor with high affinity (*K*_i_ ≈ 0.027 nM; IC_50_ ≈ 0.054 nM) and exhibits moderate antagonism at the AMY1 receptor (pKB ≈ 8.1; ~8.5 nM).^16^, ^19^ Testing of rimegepant in a panel of binding targets identified no substantial off‐target binding.^52^ Pharmacokinetically, rimegepant has an absolute oral bioavailability of approximately 64%, a median time to maximum concentration (*T*_max_) of 1.5 h, and a terminal elimination half‐life of approximately 11 h.^16^, ^52^ When rimegepant was administered with food, the *T*_max_ was delayed by approximately 1–1.5 h. A high‐fat meal reduced *C*_max_ by 42%–53% and overall exposure (AUC) by 32%–38%, whereas a low‐fat meal reduced *C*_max_ by 36% and AUC by 28%.^16^, ^52^, ^53^ The clinical relevance of the reduced rimegepant exposure observed with food administration remains unknown. See Table 1 for details.

Rimegepant is metabolized primarily by CYP3A4, with minor involvement of CYP2C9, and is a substrate for the P‐gp and BCRP efflux transporters. Excretion occurs predominantly via feces (~77%) and to a lesser extent via urine (~24%).^16^, ^52^, ^53^ No clinically significant differences in the pharmacokinetics of rimegepant were observed based on age, sex, race/ethnicity, body weight, or CYP2C9 genotype. Although no dosage adjustment is required in patients with mild, moderate, or severe renal impairment (≥15 mL/min), its use should be avoided in patients with severe hepatic impairment (Child‐Pugh class C).^52^, ^53^, ^54^, ^55^ Rimegepant is sensitive to drug–drug interactions with CYP3A4 modulators. Strong inhibitors such as itraconazole significantly increase systemic exposure, necessitating avoidance or extended dosing intervals, whereas strong inducers such as rifampin markedly reduce plasma concentrations and should be avoided altogether. Moderate inhibitors require a minimum 48‐h interval before redosing.^52^, ^53^, ^54^, ^55^ The most common adverse events in clinical trials were nausea (2%–3%) and dyspepsia (~2%), both mild in severity.^16^, ^54^ Long‐term safety data extending beyond 1 year show no evidence of hepatotoxicity or cardiovascular risk. No laboratory monitoring is required in routine practice.

### Ubrogepant

Ubrogepant was the first gepant to obtain regulatory approval for the acute treatment of migraine attacks, marking the reintroduction of small‐molecule CGRP receptor antagonists into clinical use after the discontinuation of first‐generation compounds.^15^ The recommended oral dose is 50 mg or 100 mg (both tablets available). If necessary, a second dose may be administered no sooner than 2 h after the first, with a maximum total dose of 200 mg within a 24‐h period.^56^ Structurally, ubrogepant has high selectivity toward the human CGRP receptor (*K*_i_ ≈ 0.07 nM) with 100‐fold and greater than 29,000‐fold higher affinity compared to the human AMY1 and AM2 receptors, respectively.^20^, ^57^ No significant off‐target receptor binding was reported.

Ubrogepant has a median *T*_max_ of approximately 1.5 h and a terminal elimination half‐life of 5–7 h and displays dose‐proportional pharmacokinetics within the recommended dose range. When administered with a high‐fat meal, the *T*_max_ was delayed by 2 h and resulted in a 22% reduction in *C*_max_ with no change in AUC. Ubrogepant is highly distributed (apparent volume of distribution [Vdl] = 350 L) with limited brain penetration (cerebrospinal fluid: plasma = 0.03).^20^, ^57^, ^58^ The systemic clearance occurs primarily via oxidative metabolism by cytochrome CYP3A4, with formation of two inactive glucuronide metabolites. The drug is also a substrate for P‐gp and BCRP. Approximately 42% of the administered dose is excreted in feces and 6% in urine, with minimal unchanged renal elimination.^20^, ^57^, ^58^ See Table 1 for details.

Based on population pharmacokinetic analyses, age, sex, race, and body weight did not have a significant effect on the pharmacokinetics (*C*_max_ and AUC).^20^, ^57^, ^58^ In patients with preexisting mild (Child‐Pugh class A), moderate (Child‐Pugh class B), or severe hepatic impairment (Child‐Pugh class C), ubrogepant exposure was increased by 7%, 50%, and 115%, respectively. No dose adjustment is recommended for patients with mild or moderate hepatic impairment whereas in patients with severe hepatic impairment (Child‐Pugh class C) the recommended dose is 50 mg.^20^, ^56^, ^58^, ^59^, ^60^ No dose adjustment is recommended for patients with mild or moderate renal impairment. Dose adjustment (50 mg) is recommended for patients with severe renal impairment (CLcr 15–29 mL/min). In patients with end‐stage renal disease (CLcr <15 mL/min), ubrogepant should be avoided.^56^, ^57^ Ubrogepant is sensitive to CYP3A4 modulation and therefore drug–drug interactions. Concomitant administration with the strong inhibitor ketoconazole increases systemic exposure by ~9.7‐fold, leading to contraindication, whereas strong inducers such as rifampin reduce exposure by ~78%. With moderate CYP3A4 inhibitors, the recommended maximum dose is 50 mg in 24 h.^20^, ^56^, ^57^, ^58^ The most frequent reported adverse events were nausea (2%–4%) and somnolence (~2%), no clinically significant hepatotoxicity signal was identified, and no laboratory monitoring is required in routine practice.^15^, ^57^, ^60^

### Zavegepant

Zavegepant is the most recent gepant (third generation) to enter clinical practice and the first to be developed for intranasal administration.^14^ It is approved as acute treatment. It is available as a 10‐mg nasal spray. Structurally, it retains high binding affinity for the human CGRP receptor (*K*_i_ ≈ 0.023 nM) and is engineered for rapid systemic absorption via the nasal mucosa.^14^, ^61^ Following a single intranasal dose, zavegepant achieves a median *T*_max_ of 0.5–1 h. The absolute bioavailability is approximately 5% with a terminal elimination half‐life of 6–7 h. Zavegepant undergoes metabolism primarily by cytochrome CYP3A4 and, to a lesser extent, by CYP2D6. It is also a substrate for OATP1B3 and NTCP transporters. Elimination occurs predominantly via feces, with minimal urinary clearance.^14^, ^61^, ^62^ See Table 1 for details.

Age, sex, race, ethnicity, body weight, and moderate hepatic impairment (Child‐Pugh class B) do not have a clinically significant impact on the pharmacokinetics of zavegepant. The effect of severe hepatic impairment (Child‐Pugh class C) has not been evaluated. Because renal excretion contributes minimally to zavegepant clearance, mild or moderate renal impairment (estimated creatinine clearance ≥30 mL/min) is not expected to produce clinically relevant changes in drug exposure.^14^, ^61^, ^62^, ^63^ Coadministration of a single 10‐mg dose of zavegepant with itraconazole, a strong CYP3A4 and P‐gp inhibitor, at steady state did not produce a clinically relevant change in exposure. In contrast, coadministration of a single 100 mg oral dose of zavegepant with rifampin, an OATP1B3 and NTCP inhibitor as well as a strong CYP3A inducer, at steady state increased its exposure, with AUC rising 2.3‐fold and *C*_max_ increasing 2.2‐fold. This effect reflects the combined influence of OATP1B3 and NTCP inhibition together with CYP3A enzyme induction. Therefore, zavegepant should not be used with drugs that inhibit or induce the OATP1B3 or NTCP transporters because such interactions may alter its systemic exposure.^61^, ^62^, ^63^

The most frequently reported adverse event was dysgeusia (20.5%), often described as metallic and typically transient, followed by nasal discomfort (3.7%). These effects were mild in intensity and rarely led to discontinuation.^14^, ^63^ No clinically relevant hepatotoxicity signal has been identified, and the intranasal route allows avoidance of gastrointestinal absorption issues that may occur during migraine attacks.

## BBB PERMEABILITY AND CNS CONCENTRATION OF GEPANTS

There remains considerable debate around the primary site of action of gepants, with the main open question concerning their direct effect on CNS. Although a comprehensive evaluation is outside the scope of this review (and it is reviewed elsewhere^64^), here, we summarize the pharmacological evidence of the BBB permeability of gepants and their concentration within the CNS based on preclinical and clinical data. Overall, gepants pharmacokinetics properties restrict their permeability to BBB. Indeed, their molecular weights (~530–600 Da), polar surface areas (>90 Å^2^), and relatively high polarity reduce passive diffusion across the BBB, whereas high plasma protein binding (87%–95%) and transporter‐mediated efflux via P‐glycoprotein further limits unbound drug available for CNS penetration.^18^, ^20^, ^50^, ^52^, ^59^, ^61^

Preclinical quantitative whole‐body autoradiography studies with radiolabeled gepants have demonstrated restricted CNS distribution patterns. Atogepant tissue distribution studies using [^14^C]‐atogepant in rats showed widespread distribution to peripheral tissues with concentrations similar to blood, whereas CNS tissues, eye, and bone exhibited negligible radioactivity. Consistently, a preclinical study in Sprague–Dawley rats confirmed its minimal brain penetration, with CSF/plasma AUC ratios of 0.0038 and 0.0031 and brain tissue/plasma AUC ratios of 0.0161 and 0.0157 after 5 and 20 mg/kg oral doses, respectively. Even after chronic dosing (10 and 30 mg/kg twice daily for 28 days), only mild and transient behavioral changes were observed, without withdrawal effects, even at exposures 4.7‐fold (*C*_max_) and 8.6‐fold (AUC) above the human 60‐mg dose.^18^, ^50^ Likewise, ubrogepant quantitative whole‐body autoradiography studies in rats and mice revealed minimal brain accumulation (0.006–0.12 μg*eq/g tissue in CNS regions), with radioactivity predominantly confined to peripheral tissues such as liver, kidney, and gastrointestinal tract.^20^, ^59^

Human data, although limited, align with preclinical evidence. Positron emission tomography receptor occupancy studies with telcagepant, a first‐generation gepant, demonstrated low binding in cortical regions.^65^ In particular, the tracer [^11^C]MK‐4232 was used to measure central CGRP receptor occupancy by telcagepant. Ten healthy male volunteers participated. Baseline scans showed rapid BBB penetration of the tracer, with highest uptake in the cerebellum and moderate uptake in other gray matter regions, consistent with CGRP receptor distribution. The tracer demonstrated low test–retest variability (mean, 6% ± 3%), confirming suitability for occupancy measurements.^65^ After a high, supratherapeutic oral dose of telcagepant (1120 mg), moderate central receptor occupancy (43%–58%) was observed when positron emission tomography T was performed ~3 h post‐dose, corresponding to peak plasma concentrations (16–22 μM). In contrast, the clinically effective acute migraine dose (140 mg) produced very low receptor occupancy (4%–10%) at ~2 h post‐dose. These findings indicate that central CGRP receptor blockade is minimal at therapeutically effective doses, considering that the usually required receptor occupancy for effective drugs is 60%–90%. However, the authors note that higher central receptor occupancy could potentially enhance efficacy in some patients, and further studies with more brain‐penetrant antagonists are needed to test this hypothesis, above all using currently available gepants.^65^ This pharmacokinetic–pharmacodynamic relationship indicates that efficacy can be achieved without substantial CNS exposure. Importantly, the limited CNS distribution of gepants might contribute to their favorable safety profile, reducing the risk of sedation, cognitive impairment, or mood changes often associated with centrally penetrant agents.

However, although BBB penetration is low, this does not preclude gepants from exerting limited central effects in areas with reduced barrier function, such as the trigeminal *nucleus caudalis*, area postrema, and circumventricular organs or that the very low concentration in the CNS has some minor effect. Interestingly, a randomized controlled trial demonstrated that ubrogepant is effective even during the prodromal phase (that may include symptoms such as cognitive dysfunction).^66^ This raises the question of whether its primary site of action is peripheral, with secondary central effects, as suggested for photophobia, phonophobia, or neck discomfort, or whether it also acts directly within the CNS through a primary central mechanism.^66^

## LONG‐TERM SAFETY CONSIDERATION

CGRP is not expressed exclusively in the trigeminovascular system and the CNS but has established physiological roles across multiple organ systems.^9^ This distribution raises mechanistic considerations regarding the long‐term consequences of sustained CGRP pathway blockade. However, several studies have reported an overall favorable tolerability profile of anti‐CGRP mAbs, including during relatively long‐term use of 3–5 years.^4^

Regarding gepants, post‐marketing safety data remain limited. Most available evidence derives from clinical trials (up to 52 weeks),^67^, ^68^ and short‐ to midterm observational studies^69^, ^70^, ^71^ (see paragraph below). A recent pharmacovigilance analysis based on the FDA Adverse Event Reporting System has evaluated all approved gepants,^72^ but long‐term treatment data are not yet available.

In principle, the risk associated with CGRP pathway blockade with gepants is similar to that observed with anti‐CGRP mAbs.

Briefly, CGRP has broad physiological roles across multiple organ systems. In the cardiovascular system, it acts as a potent vasodilator with protective functions during ischemic stress, raising theoretical concerns that chronic CGRP inhibition could blunt endogenous cardioprotective responses. However, clinical trials and long‐term extension studies of gepants and anti‐CGRP mAbs have not shown increased cardiovascular or cerebrovascular risk to date.^9^, ^73^, ^74^, ^75^

In the gastrointestinal system, CGRP regulates motility, visceral sensation, mucosal blood flow, and epithelial integrity, with experimental evidence supporting gastroprotective and ulcer‐healing effects. CGRP blockade may theoretically impair these mechanisms, but clinically the main observed adverse event is constipation, particularly with receptor antagonism, without consistent evidence of inflammatory bowel disease or mucosal injury.^9^, ^74^

CGRP also contributes to wound healing and inflammatory regulation by promoting angiogenesis, keratinocyte proliferation, and controlled immune responses. Although preclinical data suggest potential risks of delayed healing or altered inflammation with long‐term inhibition, such effects have not been observed in clinical trials or post‐marketing data.^9^, ^74^

Overall, despite CGRP's involvement in protective and compensatory mechanisms, current clinical evidence supports a favorable long‐term safety profile of CGRP pathway inhibition (mainly based on data with anti‐CGRP mAbs). Ongoing pharmacovigilance and long‐term observational studies remain necessary, particularly in patients with significant comorbidities or prolonged exposure and treated with gepants.

## ROLE OF GEPANTS IN MEDICATION‐OVERUSE HEADACHE

MOH is caused by the frequent use of acute medications, leading to increased headache frequency and reduced treatment responsiveness.^76^ Multiple mechanisms, including central sensitization, altered descending pain modulation, and upregulation of pronociceptive neuropeptides have been proposed to contribute in MOH pathophysiology.^76^ Preclinical studies suggest that repeated triptan or opioid exposure can increase CGRP release and trigeminal neuronal excitability.^77^ The association between MOH and gepants remains unclear due to the limited availability of clinical data. Preclinical studies in rodents show that persistent exposure to triptans and lasmiditan, but not to gepants, induces central sensitization and cutaneous allodynia, both markers of migraine chronification and potential MOH risk.^77^, ^78^, ^79^ Mechanistically, this difference may relate to the presynaptic action of triptans and ditans, which inhibit CGRP release from trigeminal afferents, repeated activation of this pathway may drive maladaptive neuroplastic changes. In contrast, gepants act post‐synaptically at the CGRP receptor, attenuating downstream signaling only when the system is active, which may result in a reduced or absent compensatory sensitization and a lower likelihood of chronicization. Clinical data also support this favorable profile: in the long‐term safety study BHV3000‐201,^80^, ^81^ patients used rimegepant as needed for up to 52 weeks. In the study, mean monthly tablet use remained almost stable, from 7.9 tablets in weeks 4–8, to 7.3 tablets in weeks 48–52, and the patients experienced stable or reduced monthly migraine days without rebound headaches or escalating drug intake. Similar findings have been reported for ubrogepant. Nonetheless, prospective, controlled trials are required to definitively assess the MOH risk profile of gepants, potentially with a higher use of tables per months.

## POTENTIAL OF COMBINING GEPANTS WITH OTHER MIGRAINE TREATMENTS

The discussion of combination strategies involving gepants in the present review is intentionally centered on pharmacological and mechanistic considerations, rather than on comparative clinical efficacy. This approach reflects the current paucity of RCTs (and other evidence at all) specifically designed to evaluate combination regimens. A comprehensive overview of available clinical evidence, including study designs, sample sizes, outcomes, and safety findings, has been recently provided in a dedicated review.^82^

### Anti‐CGRP pathway monoclonal antibodies

The concomitant use of gepants with anti‐CGRP mAbs has strong mechanistic plausibility and emerging clinical support. Anti‐CGRP mAbs, either ligand‐targeting or receptor‐targeting, achieve sustained inhibition of CGRP pathway signaling by binding their target with subnanomolar affinity and maintaining target occupancy for weeks due to elimination half‐lives of ~28–31 days.^13^ Gepants, in contrast, are small‐molecule antagonists with short half‐lives (5–11 h) that can be administered flexibly for acute or preventive purposes. Main differences are reported in Table 2. This pharmacokinetic difference means that anti‐CGRP mAbs provide a constant baseline blockade, whereas gepants can be also used transiently to block residual receptor activation during CGRP increase (i.e., as acute treatment during migraine attacks) or to increase the overall blockade of CGRP (i.e., as preventive treatment).^4^

**TABLE 2:** Key pharmacological differences between anti‐CGRP monoclonal antibodies and gepants.

	Anti‐CGRP monoclonal antibodies	Gepants
Molecular size	~150 kDa protein biologics	<600 Da small molecules
Mechanism of action	Ligand‐binding (fremanezumab, galcanezumab, eptinezumab) neutralize circulating CGRP; receptor‐binding (erenumab) prevents ligand–receptor interaction	Reversible competitive antagonism at CGRP receptor; some also block amylin 1 receptor
Administration	Subcutaneous or intravenous injection	Oral tablet/ODT or intranasal spray
Half‐life	~28–31 days	5–11 h
Metabolism	Proteolytic degradation; no CYP involvement	Primarily CYP3A4; substrates for P‐gp and BCRP
Drug–drug interactions	Minimal	Potential with strong CYP3A4/P‐gp/BCRP modulators
Dosing frequency	Monthly or quarterly	Acute, daily, or every other day
Flexibility in dosing	Low‐ sustained exposure limits rapid adjustment	High‐rapid discontinuation or titration possible
Typical clinical role	Long‐term prevention	Acute treatment and/or prevention; flexible regimens

Abbreviations: CGRP, calcitonin gene‐related peptide; CYP, cytochrome; Da, Dalton; kDa, kilodalton; ODT, orally disintegrating tablet.

Indeed, due to their smaller molecular size, gepants may reach sites inaccessible to larger mAbs. In addition, both gepants and erenumab antagonize the AMY1 receptor, offering a potential secondary mechanism of action.^4^ However, it remains uncertain whether such regimens would provide complete pathway inhibition in patients with suboptimal monotherapy response or instead increase adverse event risk without significant added benefit. The theoretical benefit is further strengthened by the pharmacodynamic distinction between ligand and receptor antagonism. Ligand‐binding anti‐CGRP mAbs reduce circulating free CGRP; however, local release in tissues with limited antibody penetration may still activate receptors, which can be blocked by gepants. Even in patients treated with erenumab, which targets the receptor, a gepant may provide additional receptor occupancy during peak CGRP release, because anti‐CGRP mAb‐receptor binding is not always immediate or complete. Evidence on combining different classes of CGRP inhibitors for migraine treatment remains limited.^28^, ^83^, ^84^, ^85^ Few studies, including a phase‐1 trial, have reported the use of gepants as acute therapy in patients already receiving anti‐CGRP mAbs (reviewed recently in Pellesi et al.^82^). Phase‐1 data indicate no pharmacokinetic interaction between ubrogepant and either erenumab or galcanezumab. Adverse events associated with both mAbs and gepants are generally uncommon and rarely severe. In addition, available studies evaluating the combination of gepants or anti‐CGRP mAbs with gepants for acute treatment have reported only a small number of mild to moderate adverse events,^28^, ^83^, ^84^, ^85^ all of which resolved spontaneously without the need for intervention.

### OnabotulinumtoxinA

OnabotulinumtoxinA (BoNT‐A) acts through a distinct but complementary mechanism.^86^ Indeed, following its uptake at peripheral nerve terminals, BoNT‐A cleaves synaptosome‐associated protein 25, inhibiting the exocytotic release of acetylcholine as well as of CGRP and possibly other neuropeptides from unmyelinated C‐fibers and reduces wide‐dynamic range neuron activation.^87^ Aδ‐fibers, which also contribute to trigeminovascular activation, are relatively unaffected by BoNT‐A presynaptic blockade.^88^, ^89^, ^90^ Gepants can antagonize CGRP receptors mainly on Aδ‐fiber–derived ligand activity and inhibit high‐threshold neuron activity. When combined in preclinical studies, BoNT‐A and gepants suppress cortical spreading depression induced sensitization of both classes of trigeminovascular neurons.^88^, ^89^, ^90^ These models suggest that combining BoNT‐A with CGRP antagonists could lead to additive inhibition of neurogenic dural vasodilation and trigeminal nociceptive firing, supporting a biological basis for synergy. Although evidence from high‐quality RCTs is not yet available, early real‐world practice patterns reported the use of combination treatment.^70^, ^91^, ^92^, ^93^ The low likelihood of pharmacokinetic drug–drug interactions, the complementary sites of action, and the absence of overlapping toxicity profiles make such combinations mechanistically and clinically sound.

### Gepants and other oral migraine treatments

There are currently no clinical studies that investigated the combination of gepants with other oral preventive treatments for migraine (including only treatment suggested in international guidelines).^82^ Gepants can be theoretically coadministered with other oral migraine therapies, both for acute and preventive use, although pharmacokinetic considerations should be considered. As small‐molecule CGRP receptor antagonists, gepants are primarily metabolized by CYP3A4 and are substrates for P‐glycoprotein and BCRP, making them susceptible to interactions with agents that inhibit or induce these pathways. However, common oral preventives, such as β‐blockers, angiotensin receptor blockers, calcium channel blockers, tricyclic antidepressants, and antiseizure medications, do not significantly affect CYP3A4 metabolism at dosages recommended for migraine treatment,^22^, ^30^ and no clinically relevant pharmacokinetic interactions with gepants and other migraine treatments have been reported to date.^23^, ^25^, ^26^, ^28^, ^29^, ^31^, ^33^, ^34^ Importantly, some trials assessing the safety and efficacy of gepants did not exclude the concurrent use of oral prophylactic medications. Clinically, gepants may be useful as acute therapy in patients on stable preventive regimens who continue to experience breakthrough migraine attacks, and as preventive agents in combination with other oral preventives in partial responders to monotherapy.

## SWITCHING AMONG GEPANTS, BETWEEN GEPANTS AND ANTI‐CGRP mAbs, AND COMBINING GEPANTS

Switching from anti‐CGRP mAbs to gepants and vice versa, could be a clinical strategy for prevention treatment when patients experience insufficient efficacy, tolerability issues, or accessibility barriers with their initial CGRP‐targeted therapy.^4^, ^13^, ^94^ Transitioning between gepants and mAbs may be generally straightforward, as no washout is required to prevent pharmacokinetic interactions; gepants are small molecules with rapid clearance, and anti‐CGRP mAbs are not metabolized by CYP enzymes or drug transporters. However, the long half‐life of anti‐CGRP mAbs (~28–31 days) means that CGRP pathway blockade may persist for several weeks after discontinuation and therefore should be considered before introducing a gepant in monotherapy. Clinical observations indicate that some patients who do not respond to anti‐CGRP mAbs may still respond to gepants (both as acute and preventive use), potentially due to differences in target site (ligand vs. receptor), administration route, tissue distribution, and ability to access anatomical compartments not easily accessible to mAbs.^4^, ^70^, ^71^, ^95^ Nevertheless, no RCTs have directly compared sequential or cross‐over strategies, and optimal timing, sequencing, and patient selection criteria remain undefined.

Currently there are no clinical data on switching among gepants for preventive or acute treatment. Theoretically, although all gepants act as CGRP receptor antagonists, they differ in elimination half‐life (ubrogepant, 5–7 h; zavegepant, 6–7 h; and rimegepant and atogepant, ~11 h), receptor binding profile (rimegepant and atogepant also antagonize the AMY1 receptor), and route of administration (oral tablet/orally disintegrating tablet or intranasal spray). As for anti‐CGRP mAbs,^13^ these pharmacological differences may translate into variable clinical responses, so lack of efficacy with one gepant does not necessarily predict nonresponse to another. Interestingly, a recent study (TANDEM study)^96^ showed that the use of atogepant 60 mg for the preventive treatment of episodic migraine and ubrogepant 100 mg as needed for the acute treatment of migraine over the 12‐week open‐label concomitant use treatment period was safe and well tolerated. These results confirm the findings of an early phase‐1 study.^27^

## REAL‐WORLD STUDIES WITH GEPANTS

Real‐world evidence on gepants for both preventive and acute use is rapidly increasing, including both patients naive or non‐responsive to prior anti‐CGRP pathway drugs.

Atogepant has demonstrated effectiveness and safety across multiple real‐world cohorts, including patients with treatment‐resistant migraine. For instance, the Italian STAR study^70^ showed a reduction of almost 7 days in monthly migraine days (MMDs) and a responder rate of 56.6% at week 12, with prior anti‐CGRP mAbs nonresponse not predicting poorer outcomes.

Consistently, another Italian study (GIANT) with 183 participants^71^ showed a mean reduction in MMDs by 6 days at week 12, with a ≥50% response rate observed in 65.9% of the overall cohort and in 52.9% of individuals with prior nonresponse to an anti‐CGRP mAb.^71^ Similar findings were reported in a Norwegian cohort^69^ where MMDs decreased by 7.1 days at week 24 and 53% of patients achieved a ≥50% response, including 47% of those with nonresponse to at least one prior anti‐CGRP treatment.

These observations are further supported by the SYNERGY study in Norway,^93^ a multicenter study in Spain^97^ and the retrospective RESCUE study conducted in the same country.^98^ The SYNERGY is a 24‐week prospective real‐world evaluation of atogepant added to BoNT‐A in CM. In this cohort, mean MMDs were reduced by 6.5 days and 45.1% of patients achieved a ≥50% response, including a substantial proportion of individuals previously exposed to insufficient responses to multiple preventive treatments.^93^ Similarly, the other multicenter observational study conducted across Spanish headache units included 251 patients who initiated atogepant after failing to respond to ≥1 anti‐CGRP mAb and had ≥ 3 months of follow‐up. At 3 months, 29.7% achieved a ≥50% response.^97^ Finally, the RESCUE study included 44 patients unresponsive to anti‐CGRP mAbs and 18.2% achieved ≥50% response status at three months.^98^

Across studies, safety and tolerability profiles were consistent, with adverse events reported ranging from 5% to 64% of patients, most commonly constipation, fatigue, nausea, and reduced appetite, whereas treatment discontinuation rates remained relatively low and no severe adverse events reported.^69^, ^70^, ^71^, ^93^, ^97^, ^98^ Real‐world studies on rimegepant as preventive treatment are currently lacking and highly expected.

Real‐world evidence also supports the effectiveness and tolerability of gepants as acute treatment in routine clinical practice.

In an early US real‐world cohort,^99^ 251 patients using ubrogepant treated a median of five attacks, with 47.6% achieving headache relief in ≥75% of treated attacks and 19.0% achieving headache freedom in ≥75% of attacks, whereas adverse events were reported in <10% of patients and no serious safety signals emerged.^99^ In the prospective COURAGE study,^85^ among patients receiving ubrogepant on top of anti‐CGRP mAbs, meaningful pain relief was achieved in 61.6% at 2 h and 80.4% at 4 h after the first treated attack, with return to normal function in 34.7% and 55.5% at the same time points, respectively. Across 1153 treated attacks, pain relief remained stable at 51.3% at 2 h and 73.5% at 4 h.^85^ In the UNIVERSE survey,^100^ >70% of ubrogepant users reported being satisfied or very satisfied with treatment, and concomitant acute medication use decreased substantially, with triptan use falling from ~40% pre‐ubrogepant to <20% during follow‐up and opioid use dropping below 5%.^100^

Finally, in a large US claims‐based study^101^ including 9909 propensity‐score–matched patients per group, 12‐month persistence was 75.8% for rimegepant versus 53.5% for oral triptans (odds ratio [OR], 2.72; 95% confidence interval, 2.56–2.90), with similarly higher persistence in CM (78.0% vs. 56.0%, OR, 2.86) and when compared specifically with sumatriptan (75.6% vs. 51.3%, OR, 2.92) or rizatriptan (75.1% vs. 55.1%, OR, 2.49).^101^

Regarding rimegepant, in an Italian cohort of 103 individuals (GAINER study), including for the first time also patients with CM, pain freedom at 2 h was achieved in 44.7% of treated attacks. Importantly, earlier administration of rimegepant during the attack was associated with a significantly higher likelihood of treatment response, further supporting the established concept that prompt intervention is critical for acute treatment effectiveness. Only mild adverse events were reported in 15.5% of attacks, most commonly fatigue.

These findings are consistent with a multicenter Greek real‐world study, in which pain freedom at 2 h was observed in approximately one‐third of treated attacks and clinically meaningful reductions in headache intensity were documented, with adverse events reported in approximately 17% of doses and no treatment discontinuations for safety reasons.

In parallel, nationwide registry data from Denmark indicate widespread uptake of rimegepant in clinical practice, predominantly among patients with prior triptan exposure and medication overuse, and suggest a reduction in acute medication overuse following initiation, although early discontinuation was frequent. Finally, in a large real‐world claims analysis, initiation of rimegepant for acute treatment was associated with substantially higher treatment persistence over 12 months compared with oral triptans, with 75.8% of rimegepant users remaining persistent versus 53.5% of triptan users, a finding consistent across specific triptans and in patients with CM.

## SITUATIONAL PREVENTION

Situational prevention represents an emerging therapeutic strategy with the aim to bridge the traditional categories of acute and preventive migraine management.^102^ A modified Delphi panel of headache experts reached strong consensus on the clinical utility of this customized approach, defined as a short‐term preventive treatment before and during time‐limited situations when migraine attack prevention is particularly desired.^102^ Unlike conventional prevention, situational prevention may target discrete vulnerability windows identified through predictable and unavoidable or planned triggers such as perimenstrual migraine, religious fasting periods, time zone changes due to flights, or anticipated stressful events.^103^

The pharmacological properties of gepants could be suited to such application; for example, the approximately 11 h half‐life with steady‐state concentrations achieved within a few days, and dose–exposure relationships linear across therapeutic ranges, ensuring predictable plasma levels during vulnerability windows.

To date, clinical experience with situational prevention remains limited and derives almost exclusively from rimegepant. Lipton et al.^104^ illustrated the approach through case studies involving time‐limited periods when migraine attack prevention was particularly useful. Notably, a daily dosing regimen was employed during high‐risk periods, recommending treatment not exceed 10 consecutive days or 18 monthly doses.^102^ More recently, an alternative every‐other‐day regimen has been explored specifically in the perimenstrual context, with 75 mg of rimegepant initiated 2 days before the expected onset of menstruation and continued through 2 days post‐onset.^105^

These preliminary findings should be interpreted as hypothesis generating rather than definitive. From a pharmacological standpoint, the principal concern with situational prevention is the risk of overmedication, as patients may take medication for anticipated attacks that would not have materialized even without treatment. Repeated unnecessary exposure could increase the incidence of adverse events and, despite reassuring medium‐term data, the long‐term consequences of anti‐CGRP treatments remain unknown.^106^ Randomized controlled trials are warranted to establish optimal patient selection, dosing protocols, and long‐term safety outcomes.

## GUIDELINES AND RECOMMENDATIONS

Because of the recent introduction of gepants and their differing availability across countries, only a few guidelines and recommendations are available on the use of gepants as acute or preventive treatments for migraine. In 2021, the *American Headache Society* (AHS) published a consensus statement on the use of newly introduced medications, recommending gepants (ubrogepant or rimegepant) as acute treatment options for migraine in patients who have either not responded to at least two oral triptans or have contraindications to or cannot tolerate triptans.^107^ The 2024 AHS position statement update on CGRP‐targeted therapies for migraine prevention included atogepant and rimegepant for EM, and atogepant for CM, as first‐line options.^108^ These therapies were recommended along with previous first‐line treatments without requiring demonstrated ineffectiveness of other classes of migraine preventive treatments. The National Institute for Clinical Excellence guidelines recommended atogepant as a preventive treatment for EM and CM in patients who have at least four monthly migraine days, only if at least three preventive drugs have failed.^109^

Recently, the *International Headache Society* (IHS) provided both practical recommendations and guidelines for the pharmacological treatment of migraine. The IHS global practice recommendations for the acute pharmacological treatment of migraine^110^ recommended gepants as an option for treating the acute attack in people with migraine for whom triptan monotherapy or combination therapy are not effective, only partially effective or not tolerated, or in patients with contraindications to triptans. The IHS global practice recommendations for preventive pharmacological treatments suggested^111^ for patients with nonresponse to multiple drugs, a switch to a different preventive treatment such as onabotulinumtoxinA, anti‐CGRP mAbs, and gepants. Additionally, atogepant is recommended alongside anti‐CGRP mAbs, onabotulinumtoxinA, and topiramate for people with CM. In the IHS pharmacological guidelines, rimegepant, ubrogepant, and zavegepant are recommended for the acute treatment of migraine attacks and rimegepant and atogepant for migraine prevention with strong strength of the recommendation and high quality of evidence.^94^, ^112^

Although guidelines are largely aligned regarding the use of gepants for acute migraine treatment, consistently positioning them as an option for patients who do not respond to, cannot tolerate, or have contraindications to triptans, greater divergence emerges when gepants are considered for preventive therapy. In this context, differences across recommendations mainly reflect variations in health care system organization, regulatory pathways, and reimbursement policies rather than conflicting interpretations of the available evidence. The AHS^108^ adopts a more permissive and patient‐centered approach, supporting earlier use of gepants for prevention without mandatory nonresponse to multiple traditional therapies, thereby facilitating timely access in systems with fewer reimbursement constraints. In contrast, the National Institute for Clinical Excellence^109^ and IHS^94^, ^112^ recommendations are more conservative, generally positioning gepants later in the preventive treatment algorithm and linking their use to ineffectiveness of prior treatments, in line with cost‐effectiveness‐driven decision‐making typical of publicly funded health care systems.

## CONCLUSION

Gepants represent a significant advancement in migraine treatment, offering selective and reversible CGRP receptor antagonism with favorable safety profiles and flexibility for both acute and preventive use. Their distinct pharmacological properties allow different treatment strategies, including combination approaches. Although current evidence supports their efficacy and tolerability, further studies are needed to define their long‐term safety and optimize combination regimens.

## AUTHOR CONTRIBUTIONS

**Luigi Francesco Iannone:** Conceptualization; methodology; validation; software; data curation; writing – review and editing; writing – original draft; supervision; investigation; project administration. **Marcello Silvestro:** Writing – review and editing; visualization; data curation. **Antonio Munafò:** Data curation; writing – original draft; visualization; writing – review and editing. **Marina Romozzi:** Writing – original draft; writing – review and editing; data curation; methodology; investigation; software. **Pierangelo Geppetti:** Writing – review and editing; supervision. **Antonio Russo:** Writing – review and editing; supervision.

## FUNDING INFORMATION

This research did not receive any specific grants from funding agencies in the public, commercial, or not‐for‐profit sectors.

## CONFLICT OF INTEREST STATEMENT

**Luigi Francesco Iannone** received financial support, consulting fees for the participation in advisory boards and support for attending meetings from: Teva, Eli Lilly, Lundbeck, Pfizer, Organon, and AbbVie; he is Associate Editor for Frontiers in Neurology and junior editor for Confinia Cephalagica, Cephalalgia and Cephalalgia Reports. **Marcello Silvestro** has received speaker honoraria from Novartis, Pfizer, AbbVie, Teva, and Eli Lilly. **Antonio Russo** has received speaker honoraria from Allergan, Lilly, AbbVie, Pfizer, Novartis, and Teva, and serves as an associate editor of Frontiers in Neurology (Headache Medicine and Facial Pain session). **Antonio Munafò, Marina Romozzi**, and **Pierangelo Geppetti** declare no conflicts of interest.

## Supporting information


**Figure S1:** PRISMA flow diagram of study selection.
