# Supplementary material for: The pharmacology of gepants in migraine: A scoping review on mechanisms, clinical applications, and combination strategies
Source: Headache. 2026 Jun 9;66(7):1628–43. doi: 10.1111/head.70114 (PMC13326972; doi:10.1111/head.70114)
Supplement: Supplementary file 1 — Figure S1: PRISMA flow diagram of study selection. [file HEAD-66-1628-s001.docx]

**The pharmacology of gepants in migraine: a scoping review on mechanisms, clinical applications, and combination strategies**

*Supplementary data*


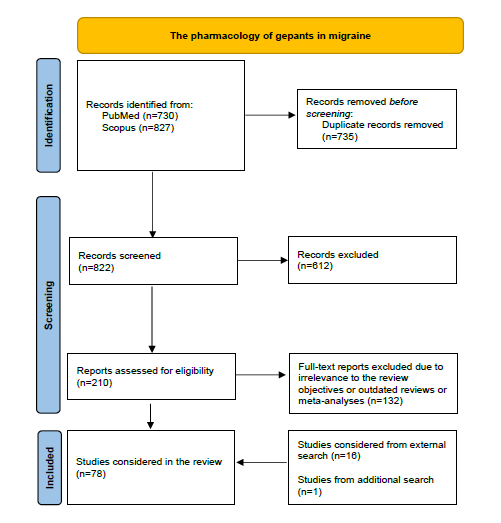


**Figure S1,** PRISMA flow diagram of study selection.
